# Cellular homologs of the double jelly-roll major capsid proteins clarify the origins of an ancient virus kingdom

**DOI:** 10.1073/pnas.2120620119

**Published:** 2022-01-25

**Authors:** Mart Krupovic, Kira S. Makarova, Eugene V. Koonin

**Affiliations:** ^a^Archaeal Virology Unit, Institut Pasteur, Université de Paris, F-75015 Paris, France;; ^b^National Center for Biotechnology Information, National Library of Medicine, Bethesda, MD 20894

**Keywords:** origin and evolution of viruses, virus capsid proteins, single and double jelly roll, virus taxonomy, exaptation

## Abstract

Viruses are a distinct type of replicators that encode structural proteins encasing virus genomes in virions. For some of the widespread virus capsid proteins and other major components of virions, likely ancestors encoded by cellular life forms are identifiable. In particular, one of the most common capsid proteins, with the single jelly-roll (SJR) fold, appears to have evolved from a particular family of cellular carbohydrate-binding proteins. However, the double jelly-roll major capsid protein (DJR-MCP), the hallmark of the enormously diverse viruses of the kingdom *Bamfordvirae* within the realm *Varidnaviria*, which includes bacterial and archaeal icosahedral viruses as well as eukaryotic giant viruses, has been perceived as a virus innovation that evolved by duplication and fusion of the SJR capsid proteins. Here we employ protein structure comparison to show that the DJR fold is represented in several widespread families of cellular proteins, including several groups of carbohydrate-active enzymes. We show that DJR-MCPs share a common ancestry with a distinct family of bacterial DJR proteins (DUF2961) involved in carbohydrate metabolism. Based on this finding, we propose a scenario in which bamfordviruses evolved from nonviral replicators, in particular plasmids, by recruiting a host protein for capsid formation. This sequence of events appears to be the general route of virus origin. The results of this work indicate that virus kingdoms *Bamfordvirae*, with the DJR-MCPs, and *Helvetiavirae* that possess two SJR-MCPs, have distinct origins, suggesting a reappraisal of the realm *Varidnaviria*.

## Significance

Viruses are the most abundant biological entities on Earth and ubiquitous parasites of cellular life forms. The general scenario for the origin of viruses involves evolution from nonviral replicators, such as plasmids and transposons, via recruitment of host proteins for virion formation. One of the most common virion core components, the double jelly-roll major capsid protein of a broad variety of viruses with double-stranded DNA genomes, so far has been thought to represent a virus innovation. However, we present evidence, obtained by protein structure comparison, that this type of virus capsid protein also evolved from a cellular ancestor, a distinct family of carbohydrate-active enzymes. These findings reinforce the chimeric scenario of virus origin.

Viruses appear to be the most abundant biological entities on Earth and the ubiquitous, obligate parasites that are associated with nearly all life forms (1–3). Unlike organisms that all possess double-stranded DNA (dsDNA) genomes, different viruses employ all forms of nucleic acids as their genetic material (4). Conceptually, viruses can be defined as a distinct type of replicators, which encode at least one protein that forms a capsid encasing the virus genome (5–7). The origin of viruses is an obviously difficult, hotly debated problem (8–16). Clearly, there was never a common ancestor of all viruses, given the absence of universal virus genes. However, each of the currently defined six virus realms (the highest rank of virus taxonomy that has no counterpart in the organismal taxonomy) (17), of which four encompass a broad variety of viruses, appears to be monophyletic (18). The scenario for the origin of viruses that currently appears to be most parsimonious involves distinct ancestries for the replication and structural modules of virus genes (16). The virus replication machineries appear to originate from the replication modules of other, capsidless replicators, such as plasmids and transposons (14–16, 19). Conceivably, given the diversity of virus replication and expression strategies, some of such replicons might trace back to the earliest stages in the evolution of life, antedating the last universal cellular ancestor (LUCA) (16, 20). By contrast, structural components of virions, and capsid proteins in particular, appear to have evolved via recruitment of functionally diverse cellular proteins, in particular, those involved in carbohydrate metabolism (16, 21). Such recruitment appears to have occurred on multiple occasions during virus evolution, resulting in the acquisition of more than 20 distinct major capsid proteins (MCPs) (21).

The MCPs have diverse structures and also widely differ in their provenance, some being encoded by an enormous variety of viruses and others restricted to narrow virus groups (21). By far the most common structural fold in MCPs is the so-called single jelly-roll (SJR) domain (22) that is also present in a broad variety of cellular proteins (21). The majority of RNA viruses in the kingdom *Orthornaviria* (one of the kingdoms in the realm *Riboviria*, the other including reverse-transcribing viruses) as well as numerous single-stranded DNA (ssDNA) viruses in the realm *Monodnaviria* possess the SJR-MCPs. By contrast, the majority of viruses in the realm *Varidnaviria* have dsDNA genomes and can have either two SJR-MCPs (kingdom *Helvetiavirae*) or the double jelly-roll (DJR)-MCPs (kingdom *Bamfordvirae*) (23). The *Helvetiavirae* includes prokaryotic viruses (24), whereas *Bamfordvirae* is a vast kingdom of viruses infecting hosts in all three cellular domains. Prokaryotic viruses with the DJR-MCPs have relatively small genomes of <20 kbp, whereas eukaryotic members of the *Bamfordvirae* have attained remarkable diversity, with their genome sizes ranging from 15 to 30 kbp in virophages (25, 26) to more than 1 Mbp in mimiviruses (27, 28). Some virus groups in this assemblage, such as poxviruses (29, 30), pithoviruses (31), and pandoraviruses (32), have either lost the DJR-MCP or evolved alternative ways of DNA packaging in the virions. For instance, the giant pandoraviruses form amphora-like rather than icosahedral virions characteristic of most varidnaviruses (32) and have apparently replaced the DJR-MCP with an inactivated and refunctionalized bacterial glycoside hydrolase (33). Poxviruses, in contrast, retain the DJR-MCP but employ it only for the formation of virion assembly intermediates, whereas the mature virions adopt a unique, brick-like shape and are constructed of distinct virus proteins (29, 30, 34, 35).

Unlike in the capsids of most smaller ssRNA and ssDNA viruses, the SJR- and DJR-MCPs of varidnaviruses are arranged on the icosahedral capsid lattice such that the axes of the two jelly-roll β-barrels are vertical with respect to the capsid surface. Accordingly, it has been suggested that the SJR-MCPs in *Helvetiavirae* have originated independently of those in *Monodnaviria* and *Riboviria* (21). By contrast, the DJR-MCP is currently thought to have evolved by fusion of the genes encoding the two SJR-MCPs of *Helvetiavirae* (23, 36, 37).

Here we employ protein structure comparison to search for potential cellular homologs of DJR-MCP. Several widespread families of cellular DJR proteins were identified, one of which (DUF2961) appears to share a direct common ancestor with the viral DJR-MCP. These findings suggest that members of the *Helvetiavirae* and *Bamfordvirae* originated independently of each other and further reinforce the chimeric scenario as the general route of virus origin.

## Results

### Identification of a Cellular DJR Protein.

To investigate the provenance of the DJR-MCPs, we searched the Protein Data Bank (PDB) database of protein structures using as queries the available DJR-MCP structures of prokaryotic viruses, including bacteriophage PRD1 (*Tectiviridae*; PDB ID: 1hx6) (38), FLiP (*Finnlakeviridae*; PDB ID: 5oac) (39), and PM2 (*Corticoviridae*; PDB ID: 2vvf) (40), and archaeal virus Sulfolobus turreted icosahedral virus (STIV; *Turriviridae*; PDB ID: 3j31) (41). The latter were chosen due to their relative simplicity and the lack of structural elaborations that are commonly found in the DJR-MCPs of larger eukaryotic viruses. Structural searches using DALI (42), in addition to the expected hits to DJR-MCPs of diverse prokaryotic and eukaryotic viruses, retrieved a structure of a hypothetical protein (PDB ID: 4kq7; BACUNI_00161) of *Bacteroides uniformis* ATCC 8492 (43). In all cases, the hits to 4kq7 were nested among the hits to bona fide viral DJR-MCPs, with highly significant Z scores above 10 (Dataset S1), indicating strong structural similarity (44). Searches seeded with 4kq7 reciprocally retrieved viral DJR-MCPs with high Z scores and, in addition, the recently characterized glycoside hydrolase (PDB ID: 7v1v; BBDE_2040) from *Bifidobacterium dentium* (45) (Dataset S1).

**Fig. 1.**
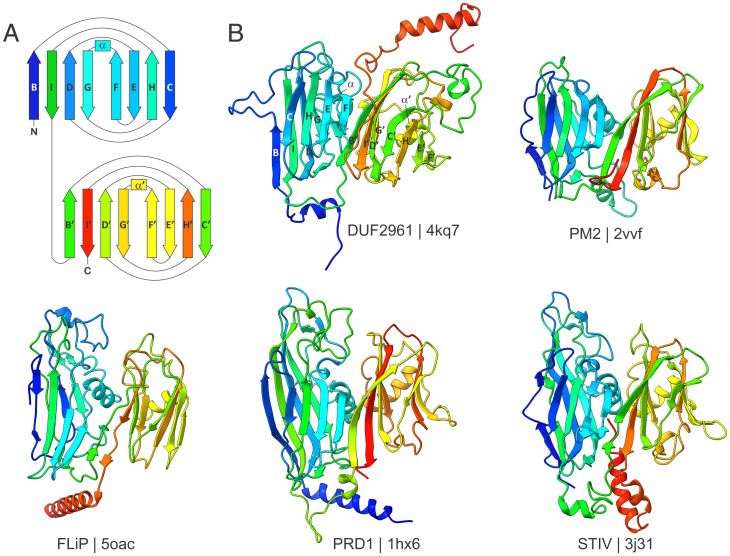
Comparison of viral and cellular DJR proteins. (*A*) Schematic diagram depicting the idealized DJR fold. Rectangles and arrows represent α-helices and β-strands, respectively, colored using a rainbow scheme (from N terminus in blue to C terminus in red). Each jelly-roll domain consists of two juxtaposed four-stranded β-sheets, BIDG and CHEF. (*B*) Comparison of the cellular DUF2961 family DJR protein with the DJR-MCPs of bacterial corticovirus PM2, tectivirus PRD1, and finnlakevirus FLiP, and archaeal turrivirus STIV. The structures are colored using the rainbow scheme, as in *A*. The PDB accessions are indicated below the corresponding structures.

Structural comparison of 4kq7 with viral DJR-MCPs confirmed that the former protein contains a DJR fold, composed of two 8-stranded β-barrels, each with the jelly-roll topology, consisting of the juxtaposed CHEF and BIDG β-sheets (Fig. 1*A*). The α-helices following the F and F′ strands, respectively, typical of the DJR-MCPs, were also conserved in 4kq7 (Fig. 1). Notably, both jelly-roll domains of 4kq7 contain an insertion of short β-hairpins upstream of the G and G′ strands of the DJR fold. At the N and C termini, the DJR of 4kq7 was bracketed by α-helices that are present in some but not all viral DJR-MCPs (Fig. 1 and *SI Appendix*, Fig. S1). A closely similar DJR fold was also found in BBDE_204, although the C-terminal α-helix was considerably longer and packed against the first (N-terminal) jelly-roll domain (*SI Appendix*, Fig. S2*A*). Thus, we conclude that 4kq7 and 7v1v are true DJR proteins that are homologous to the viral DJR-MCPs, despite negligible pairwise sequence similarity determined from structure-based alignments, which was also the case when viral MCPs were compared to each other (Dataset S1).

### A Widespread Family of Cellular DJR-MCP Homologs.

Genomes of viruses with DJR-MCPs are commonly found integrated as proviruses within bacterial and archaeal genomes (46–50). Therefore, to determine whether BACUNI_00161 is of (pro)viral or cellular origin, and to gain insight into the function of this protein and its homologs, we analyzed the domain organizations, phylogenetic distribution, and genomic neighborhoods of these proteins. In BACUNI_00161 (GenBank accession: EDO56131), the DJR domain is preceded by a predicted cleavable Sec signal sequence (Dataset S2), suggesting that the protein is exported from the cytoplasm, a feature not found in any of the viral capsid proteins. By contrast, BBDE_2040 lacked the Sec signal sequence, suggesting a cytoplasmic localization. Search against the protein families (PFAM) database showed that the DJR domain of BACUNI_00161 and BBDE_2040 belong to the DUF2961 family (PF11175) of proteins of unknown function (*E* value = 2.8e-88). BBDE_204 is an α-D-fructofuranosidase and α-D-arabinofuranosidase (45) and currently is the only experimentally characterized member of the DUF2961 family. BBDE_204 is unrelated to other known enzymes and is considered to represent a distinct family of glycoside hydrolases (45).

**Fig. 2.**
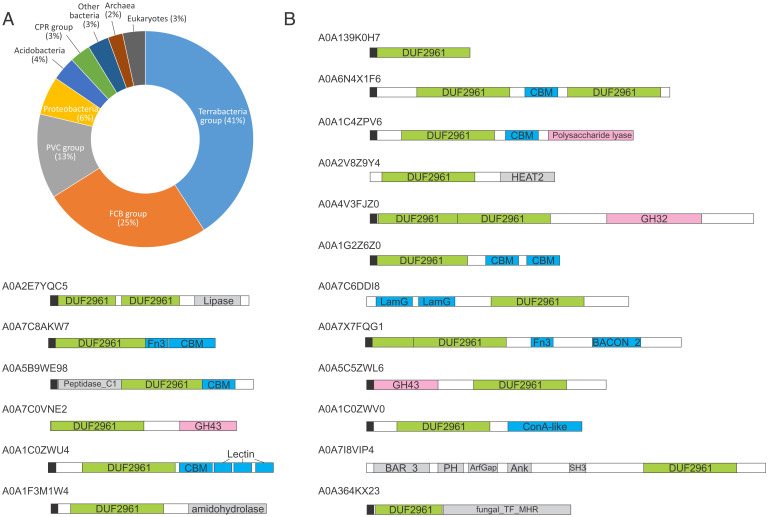
Distribution and domain organizations of DUF2961 family proteins. (*A*) Taxonomic distribution of DUF2961 family proteins in bacteria, archaea, and eukaryotes. FCB group: Fibrobacteres, Chlorobi, and Bacteroidetes; PVC group: Planctomycetes, Verrucomicrobia, and Chlamydiae; CPR group: bacteria of the Candidate phyla radiation. Detailed breakdown of taxa encoding DUF2961 family proteins is provided in Dataset S3. (*B*) Domain organizations of DUF2961 family proteins. Proteins are indicated with their UniRef identifiers and colored boxes represent protein domains, whereas black boxes represent putative signal sequences (Dataset S2). GH, glycoside hydrolase; LamG, laminin G; Fn3, fibronectin 3; BACON, *Bacteroides*-associated carbohydrate-binding often N terminal; ConA, concanavalin A; BAR, Bin-Amphiphysin-Rvs; PH, pleckstrin homology; Ank, ankyrin; SH3, SRC homology 3; fungal_TF_MHR, fungal transcription factor regulatory middle homology region.

To gain insight into the distribution and functional diversity of DUF2961 family members, we performed a jackhmmer search (*E*-value inclusion threshold of 1e-05; three iterations) queried with the BACUNI_00161 sequence against the Universal Protein Resource (UniProt) database. The retrieved homologous proteins (Dataset S3) were distributed across all three domains of life, but not viruses. The overwhelming majority of the identified homologs were from bacteria (*n* = 5,716), followed by eukaryotes (*n* = 205) and archaea (*n* = 135); the remaining 85 homologs were identified in various metagenomics datasets. In bacteria, DUF2961 family proteins are abundantly represented in the Terrabacteria supergroup (particularly, in Actinobacteria, Armatimonadetes, Chloroflexi, and Firmicutes), FCB supergroup (particularly, in the Bacteroidetes/Chlorobi group, Gemmatimonadetes, *Candidatus* Hydrogenedentes, and *Ca*. Latescibacteria), and PVC supergroup (particularly in Planctomycetes and Verrucomicrobia). In archaea, most homologs were from *Ca*. Bathyarchaeota, *Ca*. Lokiarchaeota, and Crenarchaeota, whereas the majority (77.5%) of eukaryotic homologs were from fungi (Fig. 2*A* and Dataset S3).

Although a substantial majority (91%) of the identified DUF2961 family members are single-domain proteins, similar to BACUNI_00161 and BBDE_204, variation of the domain architecture was observed as well, with the DUF2961 domain being fused to many other domains (Fig. 2*B*). In particular, DUF2961 was commonly found in combination with diverse carbohydrate-binding and cell attachment domains, including carbohydrate-binding modules (CBMs) with the jelly-roll fold, carbohydrate-binding concanavalin A, lectin and fibronectin domains, laminin G domain, and BACON domain (Fig. 2*B*). In other proteins, DUF2961 was combined with various enzymatic domains, such glycoside hydrolases of families 32 (PF00251) and 43 (PF04616), polysaccharide lyase (cd10318), GDSL-like lipase (PF13472), amidohydrolase (PF04909), and cysteine peptidase (PF00112). The experimentally verified enzymatic activity of BBDE_204 and these domain associations strongly suggest that the majority of DUF2961 members are involved in carbohydrate metabolism and/or binding. Notably, the eukaryotic homologs have a unique domain organization and likely have different functions. For instance, in certain fungi, such as *Talaromyces amestolkiae*, DUF2961 is fused to the fungal transcription factor regulatory middle homology region (cl17093), a regulatory domain found in transcription factors and centromere-binding factor 3. Some eukaryotic proteins containing the DJR domain have complex domain architectures. Thus, in marine annelid *Dimorphilus gyrociliatus*, DUF2961 is fused to the BAR domain, PH (pleckstrin homology domain; PF00169), ArfGap (GTPase-activating protein [GAP] for Arf; PF01412), ankyrin domain (PF12796), and SH3 domain (PF14604). A similar combination of domains is found in ACAP1 (ArfGAP with coiled-coil, ankyrin repeat, and PH domains protein 1), an ADP ribosylation factor (ARF) family GAP, a conserved animal protein that acts as a key component of a clathrin complex for endocytic recycling (51).

To gain further insight into the function of DUF2961 in bacteria, we analyzed the genomic neighborhoods of the corresponding proteins by extracting and annotating 471 genomic regions consisting of 10 genes upstream and downstream of the gene encoding DUF2961 domain containing protein (Dataset S4). Consistent with the results of the domain organization analysis, DUF2961 was commonly encoded in loci containing genes for various enzymes acting on carbohydrates, extracellular solute-binding protein, ABC transporters, the FGGY family of carbohydrate kinases, NAD-dependent epimerase/dehydratase, and several other proteins with diverse functions (Dataset S4). Overall, these results reinforce the conclusion that, at least in bacteria, DUF2961 family proteins function in carbohydrate metabolism either at the cell envelope or intracellularly. Notably, no virus-specific genes were identified in the vicinity of the DUF2961 genes, indicating that this protein family is not associated with proviruses, but rather, consists of bona fide cellular proteins.

### Bacterial Homologs of DJR-MCPs Form Trimers Resembling Viral Capsomers.

**Fig. 3.**
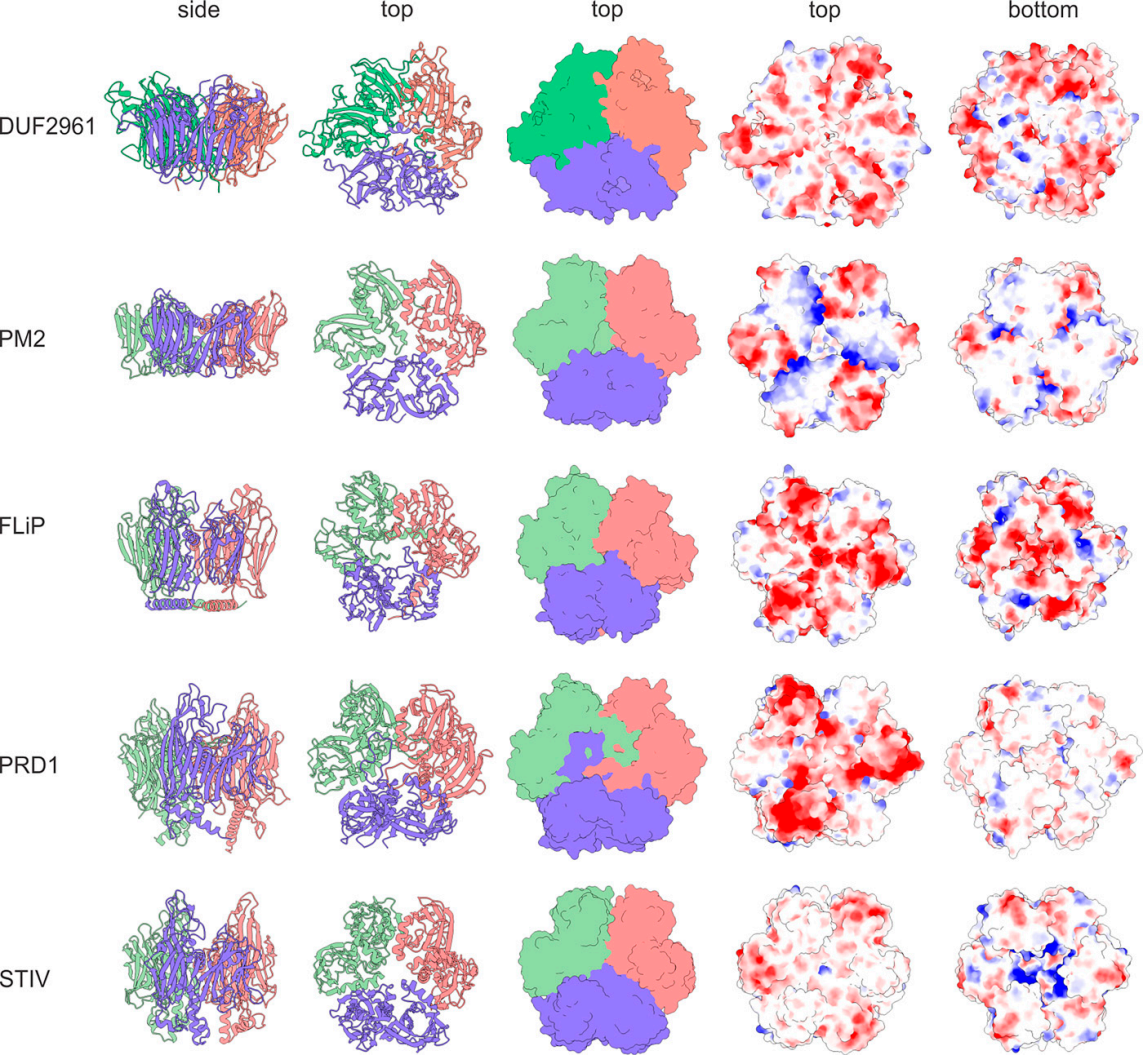
Structural comparison of the DUF2961 trimer with the trimeric capsomers of the DJR-MCPs. Individual subunits in each trimer are colored differently. The first and second columns show ribbon representations, whereas in all other columns the structures are depicted using surface rendering. In the last two columns, the structures are colored according to their electrostatic potential. The PDB accessions are the same as in Fig. 1. The C-terminal α-helix of DUF2961 is omitted for the purpose of visualization.

All viral DJR-MCPs form stable trimers with a pseudohexagonal shape, which represent capsomers, the principal building blocks of the icosahedral capsids (23, 52). Both BACUNI_00161 (4kq7) and BBDE_204 (7v1v) proteins were crystallized as tail-to-tail sandwiches of two trimers that are held together through interactions involving the C-terminal α-helices, although the exact oligomerization contacts are different in the two proteins (*SI Appendix*, Fig. S2*B*). Notably, the active site of BBDE_204 is located at the interface between the protomers, with the catalytic residues contributed by the two adjacent subunits (45). Thus, DUF2961 trimerization (and formation of higher order oligomers) might have precipitated the gain of enzymatic activity and was subsequently fixed due to selective advantage. Remarkably, the DUF2961 trimers closely resemble the capsomers of DJR-MCPs, with the three subunits intimately interacting through extensive contacts across the intersubunit interfaces (Fig. 3). The surface features, such as charge distribution, are also similar to those in the DJR-MCPs. The similarities between both tertiary and quaternary structures of DUF2961 family members and DJR-MCPs further corroborate the evolutionary relationship between these proteins.

### Other Cellular Proteins Containing the DJR Fold.

**Fig. 4.**
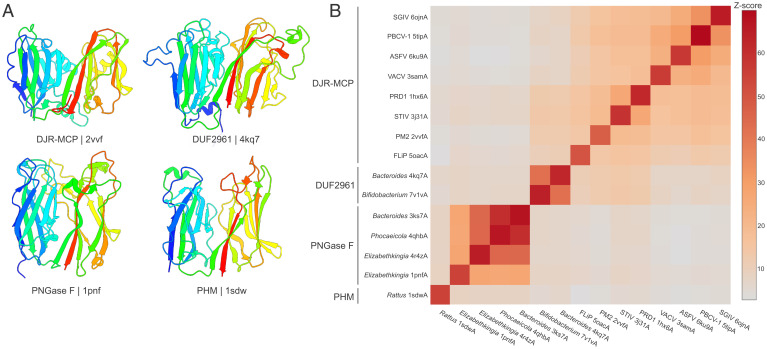
Structural comparison of viral and cellular DJR proteins. (*A*) Comparison of four families of DJR proteins: viral DJR-MCPs; DUF2961-family protein; peptide:*N*-glycosidase F (PNGase F); and peptidylglycine α-hydroxylating monooxygenase (PHM). The structures are colored using the rainbow scheme from the N terminus (blue) to C terminus (red) and the PDB identifiers are listed below the corresponding structures. The C-terminal α-helix of DUF2961 has been omitted for visualization purposes. (*B*) Relationships between cellular and viral DJR proteins. The heatmap is based on the pairwise Z score comparisons calculated using DALI. The color scale indicates the corresponding Z scores. PDB identifiers are indicated next to the corresponding rows and columns. Dataset S6 provides the complete matrix with the actual Z scores. ASFV, African swine fever virus (*Asfarviridae*); FLiP, *Flavobacterium*-infecting, lipid-containing phage (*Finnlakeviridae*); SGIV, Singapore grouper iridovirus (*Iridoviridae*); PBCV-1, Paramecium bursaria Chlorella virus 1 (*Phycodnaviridae*); PM2, Pseudoalteromonas phage PM2 (*Corticoviridae*); PRD1, Enterobacteria phage PRD1 (*Tectiviridae*); VACV, vaccinia virus (*Poxviridae*).

To search for more divergent DUF2961 homologs, we used TopSearch (53), queried with the 4kq7 structure. Consistent with the results of the DALI analysis, the best hits were to the DJR-MCPs. However, the hits to MCPs were interspersed with those to peptide:*N*-glycosidase F (PNGase F), an enzyme that cleaves the amide bond between an asparagine and oligosaccharides in N-linked glycoproteins and glycopeptides (54). Analysis of the PNGase F structures showed that they also adopt the DJR fold, with the same arrangement of the β-strands as in DUF2961 and DJR-MCPs, and, importantly, a closely similar relative orientation of the two jelly-roll subdomains (Fig. 4*A*). A notable difference between PNGases F and the other DJR proteins is the absence of the α-helices, which follow the F and F′ strands in DJR-MCPs and DUF2961. The equivalent regions are variable in the PNGases F and contain either long loops or insertions (for example, in 3ks7, F′ strand is followed by a 60-amino-acid region encompassing an extended β-hairpin, which is not present in 1pnf). Similar to DJR-MCPs, PNGases F also lack the β-hairpins found in 4kq7 upstream of the G and G′ strands.

Additional DALI searches queried with the PNGase F structure (PDB ID: 1pnf) retrieved multiple hits to functionally diverse proteins, among which peptidylglycine α-hydroxylating monooxygenase (PHM) (1sdw) was the most similar one (Z score = 8) and also had a DJR fold (Fig. 4*A*). Indeed, PNGase F and PHM constitute a PFAM clan (CL0612) and are widespread in bacteria and eukaryotes, respectively. More distant hits were to proteins, which shared with the PNGase F only one of the two jelly-roll domains. These included CBMs of diverse carbohydrate-metabolizing enzymes (for example, family 86 glycoside hydrolases [4aw7], β-glucuronidase [6xxw], cellulose synthase [2cdo], β-mannosidase [5n6u], etc.), as well as SJR capsid proteins of RNA viruses. These structural similarities are consistent with our previous conclusion that the SJR capsid proteins evolved from cellular carbohydrate-binding proteins (21). The broad distribution of DUF2961, PNGase F, and PHM family proteins in cellular organisms shows that, contrary to the previous belief, the DJR fold is not exclusive to viruses, prompting us to revisit the question of the origin of the DJR-MCPs.

### Common Ancestry of DUF2961 Family and Viral DJR-MCPs.

The cellular and viral DJR proteins, namely, DUF2961, PNGase F, PHM, and DJR-MCP, have diverged to the extent that there is no detectable pairwise sequence similarity even within some of these families (in particular, among DJR-MCPs). Thus, the relationships between the families can be analyzed only through structural comparisons. We performed an all-against-all structural comparison of DJR-MCPs, DUF2961, PNGase F, and PHM followed by average linkage clustering of the pairwise Z scores using DALI (42). In the structural similarity matrix, DUF2961 formed a cluster with DJR-MCPs, showing the closest similarity to the MCPs of prokaryotic viruses, whereas PNGase F and PHM formed two separate clusters (Fig. 4*B*). This organization of the structural similarity matrix suggests that DUF2961 could be an evolutionary intermediate between the cellular and viral DJR proteins.

**Fig. 5.**
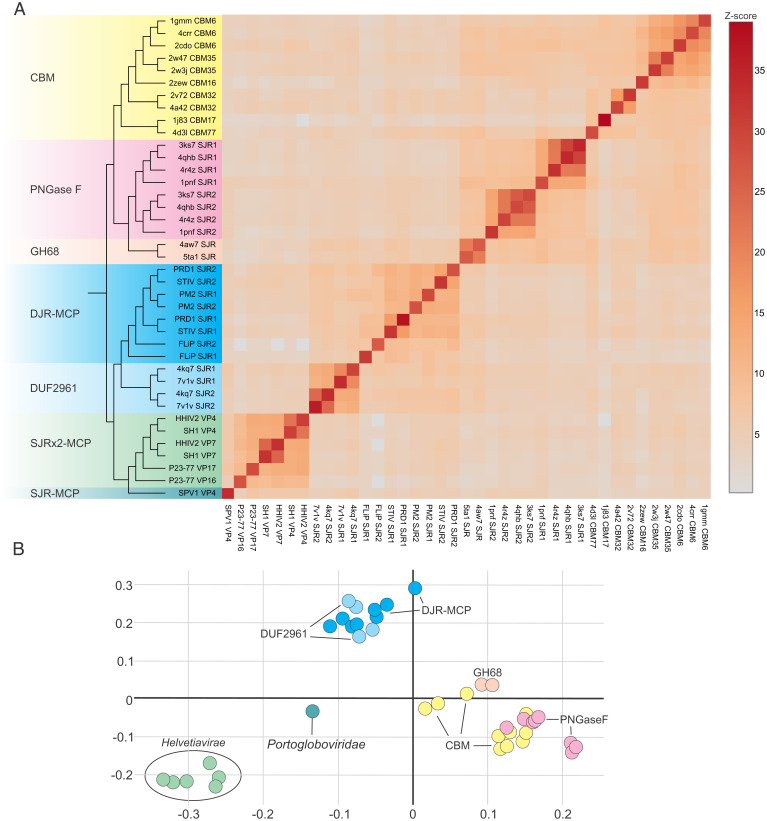
Relationships between cellular and viral SJR proteins. (*A*) The matrix and cluster dendrogram are based on the pairwise Z score comparisons calculated using DALI. Dataset S7 provides the complete matrix with the actual Z scores and PDB accession numbers. Different protein families are highlighted with different background colors on the dendrogram. The color scale indicates the corresponding Z scores. The DJR proteins have been manually split and the individual N-terminal and C-terminal SJR domains were labeled as SJR1 and SJR2, respectively. (*B*) Correspondence analysis of the cellular and viral SJR domains calculated using DALI. The data points corresponding to the SJR domains are positioned with respect to each other according to the similarity of their structural neighborhoods. The color code is the same as in *A*.

It has been previously proposed that viruses with DJR-MCPs evolved from viruses with two SJR-MCPs, namely, members of the kingdom *Helvetiavirae* (families *Sphaerolipoviridae*, *Simuloviridae*, and *Matshushitaviridae*), via fusion of the genes encoding the two SJR-MCPs (23, 36). Indeed, in helvetiaviruses, the two SJR-MCPs form homo- and heterodimers, which produce pseudohexagonal, heterohexameric capsomers that structurally resemble the homotrimeric capsomers of DJR-MCPs (37, 55–57). However, the discovery of the cellular DJR proteins prompts us to reassess the scenario for the origin of the DJR-MCPs. If the DJR-MCPs indeed evolved by fusion of two SJR-MCPs, individual jelly-roll domains of the DJR-MCPs would be expected to display closer structural similarity to the SJR-MCPs of helvetiaviruses than to other viral and cellular SJRs. By the same token, in an evolutionary tree, the helvetiaviral SJRs would be expected to form the base clade for the SJRs from the DJR-MCPs. To test this hypothesis, we focused on the DJR-MCPs of prokaryotic viruses, which display closer structural similarity to the cellular SJRs and are arguably ancestral to the eukaryotic homologs (58, 59). The DJR proteins of prokaryotic viruses as well as PNGase F and DUF2961 family proteins were split into individual SJR domains and compared to the SJR structures of helvetiaviruses, along with the CBMs of GH86 and various other carbohydrate-active enzymes. In the cluster dendrogram resulting from this expanded comparison, SJR-MCPs of helvetiaviruses and DJR-MCP viruses formed two separate branches. The two SJR domains of DUF2961 proteins were most similar to each other and comprised the sister group to the DJR-MCP SJRs (Fig. 5*A*). Placement of DUF2961 outside of the DJR-MCP assemblage is consistent with the observations that all viral DJR-MCPs can be confidently linked through sensitive profile–profile comparisons, indicative of closer evolutionary relationship, and by inference monophyly. In contrast, the relationship between the cellular and viral DJR proteins is currently detectable only at the structural level. This relationship argues against the scenario where the DUF2961 family evolved via domestication of DJR-MCPs. Further, given that the homology of all DJR-MCPs can be established through both structural and sequence comparisons, convergent evolution of viral DJR-MCPs can be effectively ruled out.

The dendrogram topology suggests that in DUF2961 the structural similarity of the two SJR domains was preserved, conceivably, due to functional constraints, such as the necessity to preserve the active site. In contrast, in the viral DJR-MCPs, exaptation of the DUF2961-like protein for the role in capsid formation was accompanied by a more pronounced divergence of one of the SJR domains. An alternative evolutionary scenario would involve independent duplication of a common ancestral SJR domain in DUF2961 and DJR-MCP.

Average linkage clustering of the Z scores consistently places the MCP of FLiP at the base of the viral clade in both the DJR-MCP (Fig. 4*B*) and individual SJR domain (Fig. 5*A*) analyses, as a neighbor to DUF2961. Notably, at the base of the helvetiaviruses SJR-MCP branch was the SJR-MCP of archaeal Sulfolobus polyhedral virus 1 (SPV1; *Portogloboviridae*) (60). This clustering supports our previous suggestion that SPV1 resembles an ancestral virus state, predating the duplication of the SJR-MCP gene in the lineage leading to the *Helvetiavirae* (59). Both FLiP in the DJR-MCP cluster and SPV1 in the SJR-MCP cluster lack genome packaging ATPases (39, 61). Thus, FLiP and SPV1 appear to represent intermediate stages of evolution of viral capsids from the respective cellular carbohydrate-binding ancestors. The requirement for ATP hydrolysis for genome packaging likely evolved in the two virus lineages independently, although the packaging ATPase potentially was captured from the same source. Indeed, helvetiaviruses and most DJR-MCP viruses encode homologous FtsK-HerA superfamily ATPases, and it was this feature that originally prompted the hypothesis on the common ancestry of these groups of viruses (62).

In the correspondence analysis, which positions data points with the most similar structural neighborhoods near each other by multidimensional scaling (42), SJRs of PNGase F and GH86 intermixed with the CBMs (Fig. 5*B*). In this analysis, the SJR of SPV1 and, to a lesser extent, those of DUF2961 also gravitated toward the CBMs in the center of the graph (Fig. 5*B*), consistent with the basal position of these structures in their respective clusters.

## Discussion

**Fig. 6.**
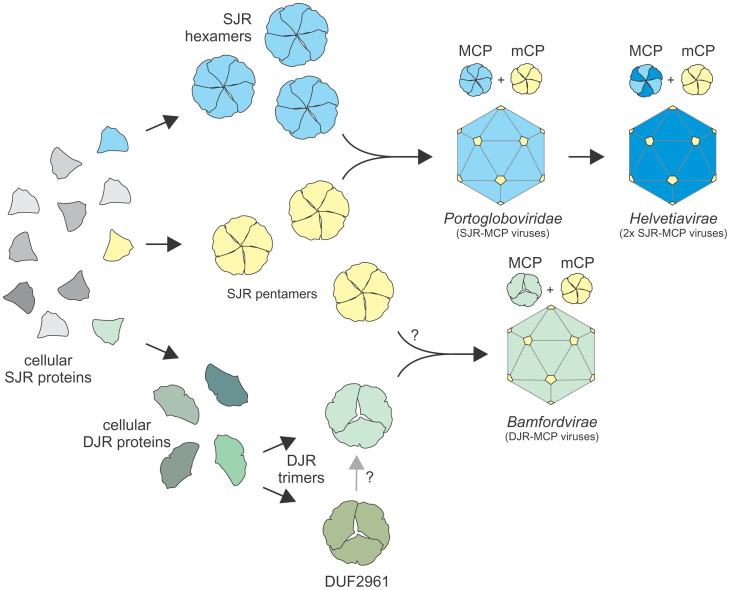
Origin and evolution of dsDNA viruses with SJR- and DJR-MCPs. SJR- and DJR-MCPs evolved from cellular proteins capable of forming hexamers and trimers, respectively, whereas minor capsid proteins (mCPs), which occupy the fivefold vertices of icosahedral capsids, have evolved from pentameric SJR proteins. Question mark denotes uncertainty. In the ancestor of *Helvetiavirae*, the gene encoding SJR-MCP was duplicated and the capsomers are heterodimers of the two SJR-MCPs.

The analysis reported here alters the parsimonious scenario for the origin of virus DJR-MCPs and, by inference, of bamfordviruses themselves. Evolution of DJR-MCPs directly from helvetiaviruses by fusion of the two SJRs is not supported by the present results. Instead, we propose that the virus lineages with the DJR-MCPs and two SJR-MCPs (bamfordviruses and helvetiaviruses, respectively) evolved independently of each other (Fig. 6). Helvetiaviruses most likely evolved from a portoglobovirus-like ancestor with a single SJR-MCP gene via a duplication of the MCP gene and acquisition of the FtsK-like genome packaging ATPase. The specific ancestor of the portoglobovirus SJR-MCP gene remains unknown. However, DALI searches queried with the structure of VP4 of SPV1 retrieve VP17 of P23-77 (Z = 7.5) as the best hit followed by XepA (PDB ID: 6i56; Z = 6.8), a protein of unknown function encoded within a lysis gene cassette of a *Bacillus* prophage (63), and several other proteins (Dataset S5), including a protein of the cupin superfamily (PDB ID: 5uqp; Z = 6.7), a highly diverse group of proteins that includes a wide variety of enzymes as well as nonenzymatic seed storage proteins (64). Notably, XepA forms pentamers (63), which resemble capsomers located at the fivefold vertices of the icosahedral capsids of portogloboviruses and varidnaviruses (37, 41, 55, 60). Cupins also form diverse homooligomers (64), suggesting that the inherent predisposition of cellular SJR proteins for oligomerization is one of the features that underlie the selection of this particular structural fold for virus capsid formation. In DALI searches queried with the MCP of portoglobovirus SPV1 or both MCPs of helvetiavirus P23-77, hits to homologous SJR-MCPs were followed by those to various cellular SJR proteins, but DJR-MCPs were not recovered in the top 10 best hits for any of the three MCPs (Dataset S5), further supporting distinct origins of helvetiaviruses and bamfordviruses.

The pseudohexagonal trimer formed by DUF2961 is remarkably similar to the capsomers of DJR-MCP viruses (Fig. 3), strongly suggesting that, contrary to the previous hypothesis, duplication and fusion of the SJR domains occurred in cellular organisms and predates the origin of DJR-MCPs. We propose that DJR-MCPs shared the most recent common ancestor with cellular DUF2961 family proteins rather than evolving from the SJRs of helvetiaviruses (Fig. 6). Alternatively, DJR-MCPs could evolve directly from the DUF2961 family, but due to scarce structural data available for DUF2961 proteins, this possibility is not currently supported by the structural dendrograms where the two groups of proteins comprised sister clades (Figs. 4*B* and 5*A*). The topology of the cluster dendrogram obtained by structural comparison of the individual SJRs comprising DUF2961 and viral DJR-MCPs along with SJR proteins is also compatible with the possibility that duplication of an ancestral SJR occurred independently in DUF2961 and DJR-MCPs.

Structural comparisons suggest that FLiP-like viruses, which lack the genome packaging ATPase and have small, simply organized genomes, are ancestral to other DJR-MCP virus groups, recapitulating the evolutionary trajectory of helvetiaviruses. Notably, FLiP-like viruses are not the only group of DJR-MCP viruses lacking the genome packaging ATPases. Another expansive group of such viruses has been discovered by metagenomics data analysis (47), and some of these have been subsequently shown to be associated with Asgard archaeal hosts (65). As in the case of helvetiaviruses, the packaging ATPase was a subsequent addition in the evolution of DJR-MCP viruses. Notably, the capsid organization of corticovirus PM2 is closely similar to that of FLiP, both being built on the T = 21 icosahedral lattice (39, 40), which is not found in any other virus. Although FLiP and corticovirus genomes are circular ssDNA and dsDNA molecules, respectively, both replicate via the rolling-circle mechanism and encode plasmid-like replication initiation endonucleases, albeit of different families (14, 39).

The emerging scenario for the origin of DJR-MCP viruses closely follows that proposed for other virus groups (14, 16), whereby the replication module evolved from preexisting nonviral replicons, such as plasmids, whereas the structural module is derived from cellular proteins that were exapted as capsid proteins and other virion components. Our understanding of the deep evolutionary connections in the virosphere has evolved dramatically over the past few years, a development that has already transformed the virus taxonomy (17, 18). Our current results suggest that, contrary to the previous hypotheses, the dsDNA virus kingdoms *Helvetiavirae* and *Bamfordvirae* are not monophyletic, that is, have distinct origins. Thus, revision of the realm *Varidnaviria* seems to be due. The continuing accumulation of sequence and especially structural data on cellular and viral proteins is bound to entail further refinement of the scenarios of the origin and evolution of each of the major groups of viruses, and the corresponding changes in virus taxonomy.

## Materials and Methods

All viral and cellular protein structures were downloaded from the PDB database (66). Protein structure-based searches were performed using the DALI server (42). Structural similarities between cellular and viral proteins were evaluated based on the DALI Z score, which is a measure of the quality of the structural alignment. Z scores above 2, i.e., two SDs above expected, are usually considered significant (44). The relevance of the matches was evaluated further by visual inspection of structural alignments between the compared proteins. Structural homologs were additionally searched for using the TopSearch server (https://topsearch.services.came.sbg.ac.at/) (53). Structural similarity matrices and correspondence analysis from all-against-all structure comparisons as well as corresponding dendrograms were obtained using the latest release of the DALI server (67). Structures were aligned using the MatchMaker algorithm implemented in University of California, San Francisco (UCSF) ChimeraX (68) and were visualized using the same software.

Sequences homologous to BACUNI_00161 (GenBank accession: EDO56131) were collected by running three iterations of jackhmmer (69) against the UniProt protein sequence database (70) with the *E*-value inclusion threshold of 1e-05. The phyletic distribution of the DUF2961 family proteins as well as the diversity of their domain organizations were retrieved from the results of the jackhmmer and further refined using HHpred (71). Signal peptides were predicted using SignalP v5 (72) and predictions with the likelihood higher than 0.8 were considered significant. For the DUF2961 family proteins from complete genomes available in the RefSeq database (March 2019) (73), genomic neighborhoods including 10 genes upstream and downstream of the gene encoding a DUF2961 family protein were extracted and annotated using PSI-BLAST (74) search against conserved domain database (CDD) profiles (75).

## Supplementary Material

Supplementary File

Supplementary File

Supplementary File

Supplementary File

Supplementary File

Supplementary File

Supplementary File

Supplementary File

## Data Availability

All study data are included in the article and/or supporting information.
