# Supplementary material for: Cellular homologs of the double jelly-roll major capsid proteins clarify the origins of an ancient virus kingdom
Source: Proc Natl Acad Sci U S A. 2022 Jan 25;119(5):e2120620119. doi: 10.1073/pnas.2120620119 (PMC8812541; doi:10.1073/pnas.2120620119)
Supplement: Supplementary File [file pnas.2120620119.sapp.pdf]

SUPPLEMENTARY INFORMATION

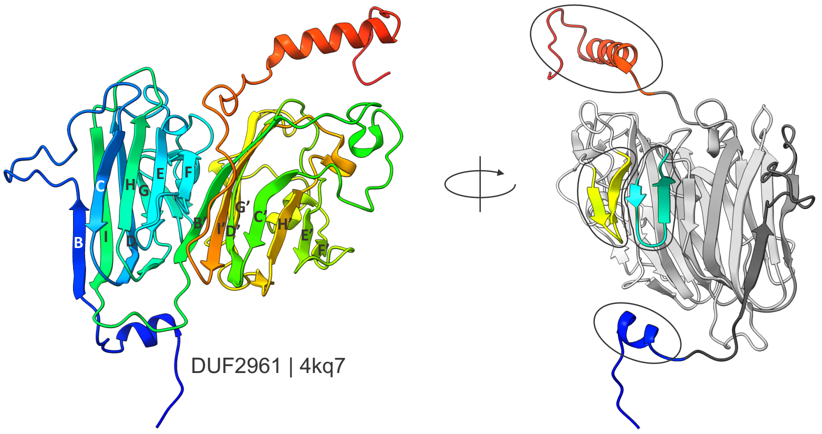

Figure S1. Different views of the structure of BACUNI_00161, the prototype of DUF2961 family. The structure on the left is colored using the rainbow scheme from the N-terminus (blue) to C-terminus (red). The β-strands are named according to the commonly used nomenclature (see Figure 1A for details). The view on the right highlights the structural elements specific to BACUNI_00161. The latter are outlined and are depicted in color, whereas the rest of the structure is shown in greyscale.

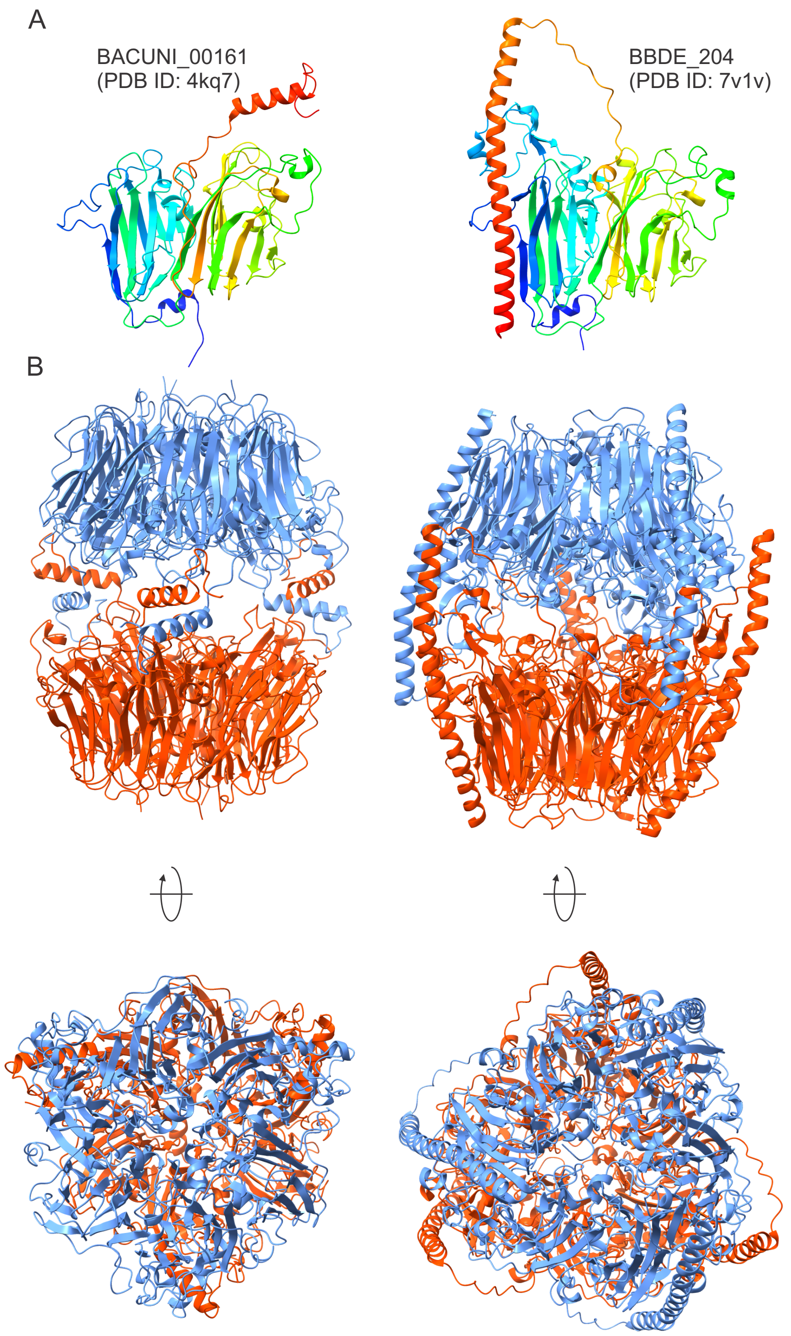

Figure S2. The structures of DUF2961 family proteins. A. Comparison of BACUNI_00161 (left) and BBDE_204 (right). The structure on the left is colored using the rainbow scheme from the N-terminus (blue) to C-terminus (red). B. Comparison of the crystallized biological assemblies of BACUNI_00161 (left) and BBDE_204 (right). Depicted are two trimers which form tail-to-tail hexamers stabilized through interactions involving the corresponding C-terminal α-helices. The two trimers in each hexamer are colored red and blue, respectively. PDB identifiers: 4kq7 (BACUNI_00161) and 7v1v (BBDE_204).
